# Growth of the inferior cavopulmonary pathway in patients with a lateral tunnel fontan connection: quantification from serial magnetic resonance images

**DOI:** 10.1186/1532-429X-14-S1-P114

**Published:** 2012-02-01

**Authors:** Maria Restrepo, Lucia Mirabella, Elaine Tang, Christopher M Haggerty, Mark A Fogel, Anne Marie Valente, Doff B McElhinney, Ajit P Yoganathan

**Affiliations:** 1 Department of Biomedical Engineering, Georgia Institute of Technology and Emory University, Atlanta, GA, USA; 2 School of Chemical and Biomolecular Engineering, Georgia Institute of Technology, Atlanta, GA, USA; 3 Division of Cardiology, Children’s Hospital of Philadelphia, Philadelphia, PA, USA; 4 Department of Cardiology, Children’s Hospital Boston, Boston, MA, USA

## Background

Single ventricle heart defects affect 2 per 1000 live births in the US and are lethal if left untreated. The Fontan procedure used to treat these defects consists of a series of palliative surgeries to create the total cavopulmonary connection (TCPC) which bypasses the right heart. In the last stage, the inferior vena cava (IVC) is connected to the pulmonary arteries (PAs) using one of two main approaches: an extracardiac conduit (EC), where a synthetic graft is used as the conduit; and the lateral tunnel (LT), where the atrial wall is used along with a synthetic patch to create the pathway. The LT pathway is anticipated to grow in the long term because it is formed partially with native atrial tissue, as opposed to the EC that retains its original size (contains only synthetic material); however, growth of LT pathways has not been systematically quantified. The objective of this work is to quantify cavopulmonary pathway growth from serial MR images of LT patients.

## Methods

Cardiac magnetic resonance (CMR) steady-state free precession cine axial image stacks (N~=45; pixel size~=1mm*1mm; slice thickness~=5mm) were analyzed. The anatomy was reconstructed using state of the art techniques developed in our research group; the inferior cavopulmonary pathway was isolated by cutting above the inferior venous confluence and before the PAs (figure). A geometrical analysis to quantify growth was performed using the Vascular Modeling Toolkit (VMTK). Results were normalized to changes in body surface area (ΔBSA=0.43±0.14m2) in order to compare changes occurring in different patients over different time spans. The metrics were compared to an EC conduit (n=1) as a control.

**Figure 1:**
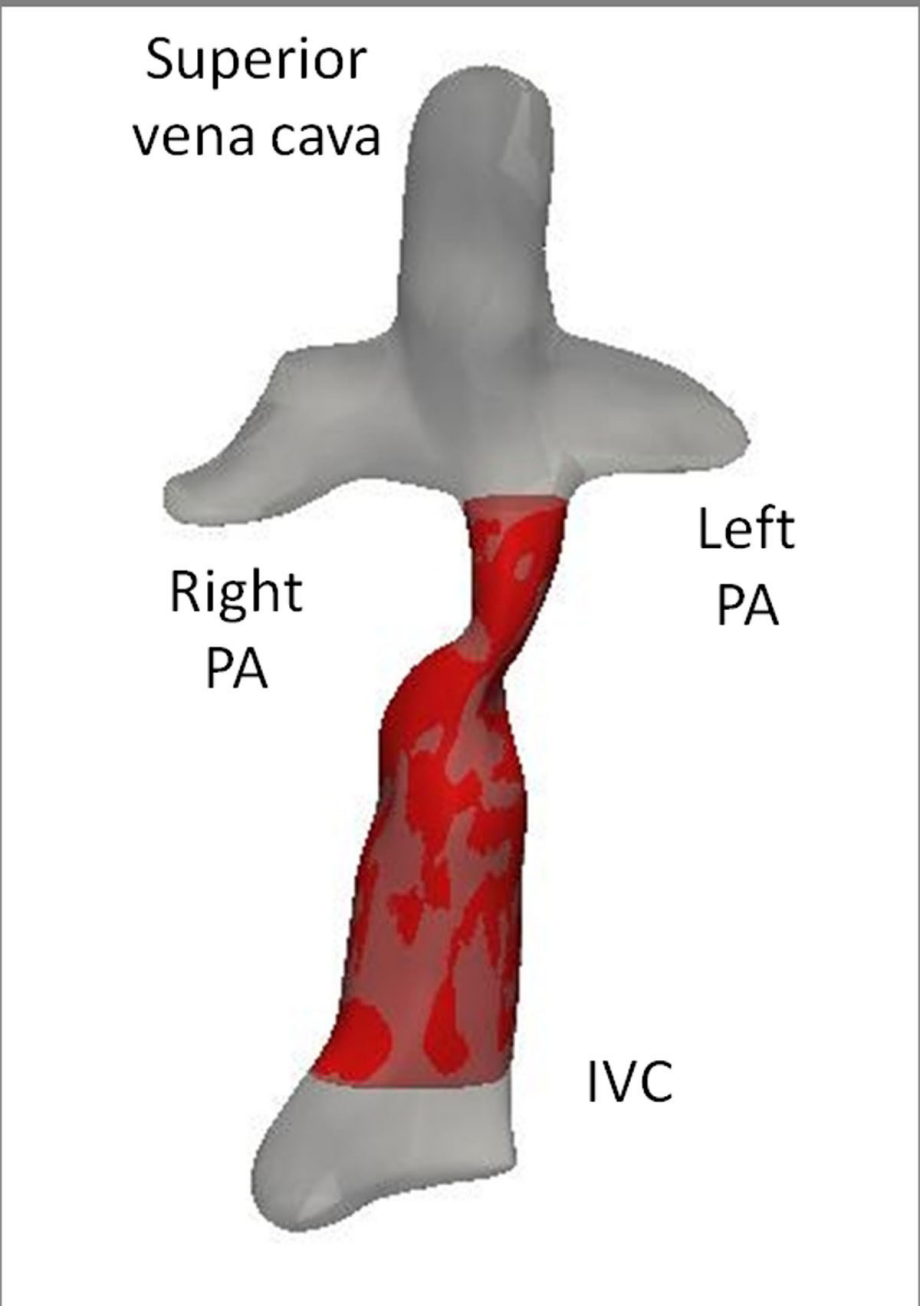
Reconstructed patient-specific anatomy (gray) and isolated lateral tunnel conduit (red).

## Results

Imaging data from 6 patients with a completed TCPC were analyzed. The time interval between subsequent CMR scans was 4.71±1.79 years. For the LT conduits considerable changes were observed between scans in the diameter, cross sectional area and length (measured along centerline), while small changes were observed in the EC (Table 1). Geometrical relationships such as shape factor (ratio between minimum and maximum diameter) and tortuosity (ratio of the length of the vessel to the shortest distance between its ends) presented small changes for both cases.

**Table 1:** Patient information and geometrical analysis summary

	LT (n=6)	EC (n=1)
Age (years) at 1st CMR	8.72±3.74	8
BSA (mm2) at 1st CMR	0.93±0.24	0.69
Age (years) at 2nd CMR	12.75±2.26	13
BSA (mm2) at 2nd CMR	1.36±0.19	1.72
Δ Mean Diameter (mm/m2)	10.56±2.31	1.98
Δ Minimum Diameter (mm/m2)	6.84±7.27	0.31
Δ Maximum Diameter (mm/m2)	17.36±5.89	3.31
Δ Area (mm2/m2)	330.97±146.99	56.61
Δ Length (mm/m2)	9.04±17.45	4.20
Δ Shape factor (m-2)	-0.13±0.33	-0.07
Δ Tortuosity (m-2)	0.01±0.06	-0.01

All variables from the geometrical analysis are normalized by BSA

## Conclusions

Serial CMR images were used to non-invasively compute the geometrical parameters in patients with LT, where changes were observed in the transverse and longitudinal direction. These results show the growth potential of the LT conduit in the long term, which will be important to consider when choosing the IVC connection type.

## Funding

National Heart, Lung, and Blood Institute Grants #HL67622 and #HL098252.

